# Lack of conspicuous sex‐biased dispersal patterns at different spatial scales in an Asian endemic goose species breeding in unpredictable steppe wetlands

**DOI:** 10.1002/ece3.6382

**Published:** 2020-06-27

**Authors:** Qin Zhu, Iderbat Damba, Qingshan Zhao, Kunpeng Yi, Nyambayar Batbayar, Tseveenmyadag Natsagdorj, Batmunkh Davaasuren, Xin Wang, Sonia Rozenfeld, Sachiko Moriguchi, Aibin Zhan, Lei Cao, Anthony D. Fox

**Affiliations:** ^1^ School of Life Sciences University of Science and Technology of China Hefei China; ^2^ State Key Laboratory of Urban and Regional Ecology Research Center for Eco‐Environmental Sciences Chinese Academy of Sciences Beijing China; ^3^ University of Chinese Academy of Sciences Beijing China; ^4^ Ornithology Laboratory Institute of Biology Mongolian Academy of Sciences Ulaanbaatar Mongolia; ^5^ Wildlife Science and Conservation Center of Mongolia Ulaanbaatar Mongolia; ^6^ Bird Ringing Centre of Russia Institute of Ecology and Evolution Russian Academy of Sciences Moscow Russia; ^7^ Faculty of Veterinary Science Nippon Veterinary and Life Science University Tokyo Japan; ^8^ Key Laboratory of Environmental Biotechnology Research Center for Eco‐Environmental Sciences Chinese Academy of Sciences Beijing China; ^9^ Department of Bioscience Aarhus University Aarhus Denmark

**Keywords:** Dauria area, genetic structure, microsatellites, molt migration, sex‐biased dispersal, Swan Goose

## Abstract

Dispersal affects the spatial distribution and population structure of species. Dispersal is often male‐biased in mammals while female‐biased in birds, with the notable exception of the Anatidae. In this study, we tested genetic evidence for sex‐biased dispersal (SBD) in the Swan Goose *Anser cygnoides*, an Asian endemic and IUCN vulnerable species, which has been increasingly restricted to breeding on Mongolian steppe wetlands. We analyzed the genotypes of 278 Swan Geese samples from 14 locations at 14 microsatellite loci. Results from assignment indices, analysis of molecular variance, and five other population descriptors all failed to support significant SBD signals for the Swan Goose at the landscape level. Although overall results showed significantly high relatedness within colonies (suggesting high levels of philopatry in both sexes), local male genetic structure at the 1,050 km distance indicated greater dispersal distance for females from the eastern sector of the breeding range. Hence, local dispersal is likely scale‐dependent and female‐biased within the eastern breeding range. These findings are intriguing considering the prevailing expectation for there to be female fidelity in most goose species. We suggest that while behavior‐related traits may have facilitated the local genetic structure for the Swan Goose, several extrinsic factors, including the decreasing availability of the nesting sites and the severe fragmentation of breeding habitats, could have contributed to the absence of SBD at the landscape level. The long‐distance molt migration that is typical of goose species such as the Swan Goose may also have hampered our ability to detect SBD. Hence, we urge further genetic sampling from other areas in summer to extend our results, complemented by field observations to confirm our DNA analysis conclusions about sex‐specific dispersal patterns at different spatial scales in this species.

## Graphical abstract

We analyzed the genotypes of 278 Swan Geese samples from 14 locations at 14 microsatellite loci. We found no significant SBD signals at the landscape level, but local dispersal is likely scale‐dependent and female‐biased within the eastern breeding range. We urge further genetic sampling from other areas in summer to extend our results, complemented by field observations to confirm our DNA analyses conclusions about sex‐specific dispersal patterns at different spatial scales in this species.

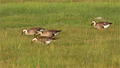

## 1 INTRODUCTION

Dispersal can be defined as the movement of an organisms from its natal place to its first breeding site (natal dispersal) or from one breeding area to a subsequent site (breeding dispersal; Gauffre, Petit, Brodier, Bretagnolle, & Cosson, 2009). Such dispersal can be affected by species ecology, weather, geography, and behavior (e.g. mobility and social organization) and can potentially lead to profound consequences for the genetic structure of populations (Newton, 2008). Dispersal entails costs, for instance energetic costs of movement (Bowler & Benton, 2005), increased exposure to predators and unfamiliar environments (Rivera, Gardenal, & Chiaraviglio, 2006), failure to find a suitable settlement site (Li & Kokko, 2019), and hostile behaviors of resident individuals toward new potential incomers (Asensio, Korstjens, Schaffner, & Aureli, 2008). Despite potential costs, dispersal can (a) reduce competition for limited resources (“resource‐competition hypothesis”; Greenwood, 1980; Wang, Lane, & Ding, 2012), (b) reduce local competition for potential mates (“local mate competition hypothesis”; Prugnolle & de Meeus, 2002), (c) avoid inbreeding with relatives (“inbreeding avoidance hypothesis”; Aharon‐Rotman et al., 2017; Bengtsson, 1978; Blyton, Banks, & Peakall, 2015; Pusey, 1987), and (d) promote cooperative breeding through kin selection (“cooperative behaviour among kin”; Gauffre et al., 2009). Factors associated with all four hypotheses often interact in nature (Starrfelt & Kokko, 2012), ultimately determining the propensity (and distance) of individuals to disperse. However, when the balance of selection differs between genders, dispersal is likely to be sex‐biased.

Males generally disperse more frequently and further than females in mammals (Gauffre et al., 2009; Li & Kokko, 2019), whereas the opposite pattern tends to hold for most bird species (Dobson, 2013; Greenwood, 1980, 1987). In socially monogamous birds with a “resource defense” mating system (Mabry, Shelley, Davis, Blumstein, & Van Vuren, 2013), familiarity with local resources has been hypothesized to be critical for males, which explains the prevalence of female‐biased dispersal in these species (Greenwood, 1980). However, the Anatidae have long been recognized as the exception to avian female‐biased dispersal, with many species having “mate defense” mating systems (Clarke, Sæther, & Roskaft, 1997; Greenwood, 1980; Wolff & Plissner, 1998). Among Anatidae, lifetime pair‐bond duration is common among swans, geese, and whistling ducks, with pairs returning together to previous breeding sites until death or separation, in which cases, females tend to exhibit breeding philopatry whereas males rarely do (Rohwer & Anderson, 1988).

Investigating empirical trends in dispersal patterns across closely related species must account for phylogeny and shared evolutionary history (Mabry et al., 2013; Perrin & Mazalov, 1999). However, phylogenetic independence of sex‐biased dispersal (SBD) has been documented by several studies across closely related species (Durand et al., 2019; Hammond, Handley, Winney, Bruford, & Perrin, 2006). The diversity of SBD patterns illustrated by these studies demonstrates the complexity of SBD evolution rather than being a product of phylogenetic inertia (Trochet et al., 2016). Generally, species characterized by SBD will exhibit different patterns when utilizing markers that differ in patterns of inheritance (Scribner et al., 2001). Using this line of evidence, studies showed that SBD was absent in the Greylag Goose *Anser anser* (Pellegrino, Cucco, Follestad, & Boos, 2015). However, male‐biased dispersal (MBD) was found in some other species, including the Lesser White‐fronted Goose *Anser erythropus* (Ruokonen, Aarvak, Chesser, Lundqvist, & Merilä, 2010), European Goosander *Mergus merganser merganser,* and North American Common Merganser *Mergus merganser americanus* (Peters, Bolender, & Pearce, 2012). In these species, there was no apparent relationship between social mating system and SBD when taking account of the effects of phylogeny (Mabry et al., 2013). For these reasons, SBD does not seem to constitute a general feature that we can determine based on the behavioral or ecological traits of closely related species.

**FIGURE 1:**
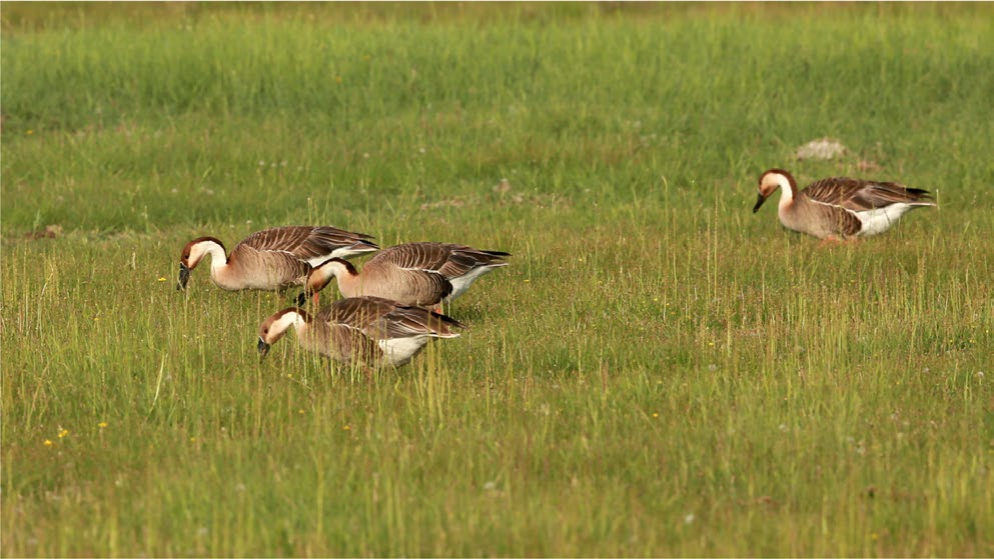
Swan Goose *Anser cygnoides* in Inner Mongolia, China. Photograph credit: Geriletu Zhao (Inner Mongolia Normal University, Inner Mongolia, China)

The Swan Goose *Anser cygnoides* is a globally vulnerable species (Figure 1) breeding in Russia, Mongolia, and China, which overwinters now almost exclusively in China, largely restricted to the Yangtze floodplain (BirdLife International, 2019). Recent breeding range contraction and fragmentation (Fox & Leafloor, 2018), low reproductive output (Goroshko, 2004), low adult survival rate (Choi et al., 2016), and declining abundance (Zhang et al., 2011) justify concern for the future effective conservation of the species. Recent assessments recognized four discrete Swan Goose breeding areas (Fox & Leafloor, 2018), emphasizing the increasing divergence between “inland” (all geese except those breeding in Far East Russia) and “coastal” (those breeding in Far East Russia) breeding populations based on earlier ring recovery data (Poyarkov, 2005) and mtDNA analyses (Poyarkov, Klenova, & Kholodova, 2010). Moreover, the persistence of biparental care and parent–offspring cohesion among geese throughout the first winter (Robertson & Cooke, 1999; Warren, Fox, Walsh, & O'Sullivan, 1993), pairing away from breeding areas and long‐term pair bonds, would suggest that SBD is not a feature of this population.

The core breeding area of the Swan Goose, the Mongolian Plateau steppe region, has entered the dry phase of a prolonged drought cycle in the last 20–30 years. Increasing climatic extremes (John et al., 2016; Pederson, Hessl, Baatarbileg, Anchukaitis, & Di Cosmo, 2014), falling annual precipitation (John et al., 2016; Liu et al., 2013; Zhou, Yamaguchi, & Arjasakusuma, 2018), and landscape effects of socioeconomic human activities (Hilker, Natsagdorj, Waring, Lyapustin, & Wang, 2014; Liu et al., 2013) have caused wetland loss and rapid lake shrinkage across the region (Tao et al., 2015). Since changing environments undoubtedly affect population demography of long‐distance migratory birds (Eichhorn, Drent, Stahl, Leito, & Alerstam, 2009; Fox et al., 2005; Rakhimberdiev et al., 2011), it is important to assess the level of connectivity among geographical populations to understand the resilience of a species to environmental fluctuations. A fundamental property that potentially shapes such resilience is SBD, which is difficult to predict in the Swan Goose, given differences among investigated related goose species (Pellegrino et al., 2015; Ruokonen et al., 2010), especially given the heavy reliance of Swan Geese upon, and site loyalty to natural wetlands, especially in winter (Yu et al., 2017).

In this study, we use biparentally inherited microsatellite markers to determine SBD in wild Swan Goose populations. Female‐biased natal and breeding site fidelity, and the timing and process of pair‐bond formation are thought to be among the most important factors regulating the magnitude and direction of gene flow in waterfowl (Scribner et al., 2001). A recent study also highlighted the importance of other factors (e.g. parental care; Trochet et al., 2016) when considering the evolution of SBD. The long‐term genetic monogamy (Toft & Wright, 2015) and high breeding philopatry of both sexes (Rohwer & Anderson, 1988) would lead us to predict that both male and female Swan Geese tend to return to familiar territories to breed. Extended parent–offspring bonds (Blackmore & Heinsohn, 2015; Rohwer & Anderson, 1988) and associated transmission of social behaviors (e.g. assortative pairing and sexual imprinting; Ely, Wilson, & Talbot, 2017) typical of geese species would strengthen family cohesion as well as transmit traits associated with mate choice (e.g. assortative pairing and sexual imprinting) across generations, promoting the maintenance of the family group across breeding and wintering grounds. As a result, in the face of an increasingly fragmented breeding range, there will be a risk of increasingly limited dispersal for both males and females. Therefore, we expected no SBD in the Swan Goose, a trait not previously investigated in this species, when we undertook the following study of SBD based on genetic evidence sampled from across the breeding range of the species.

## 2 MATERIALS AND METHODS

### 2.1 Field sampling

**FIGURE 2:**
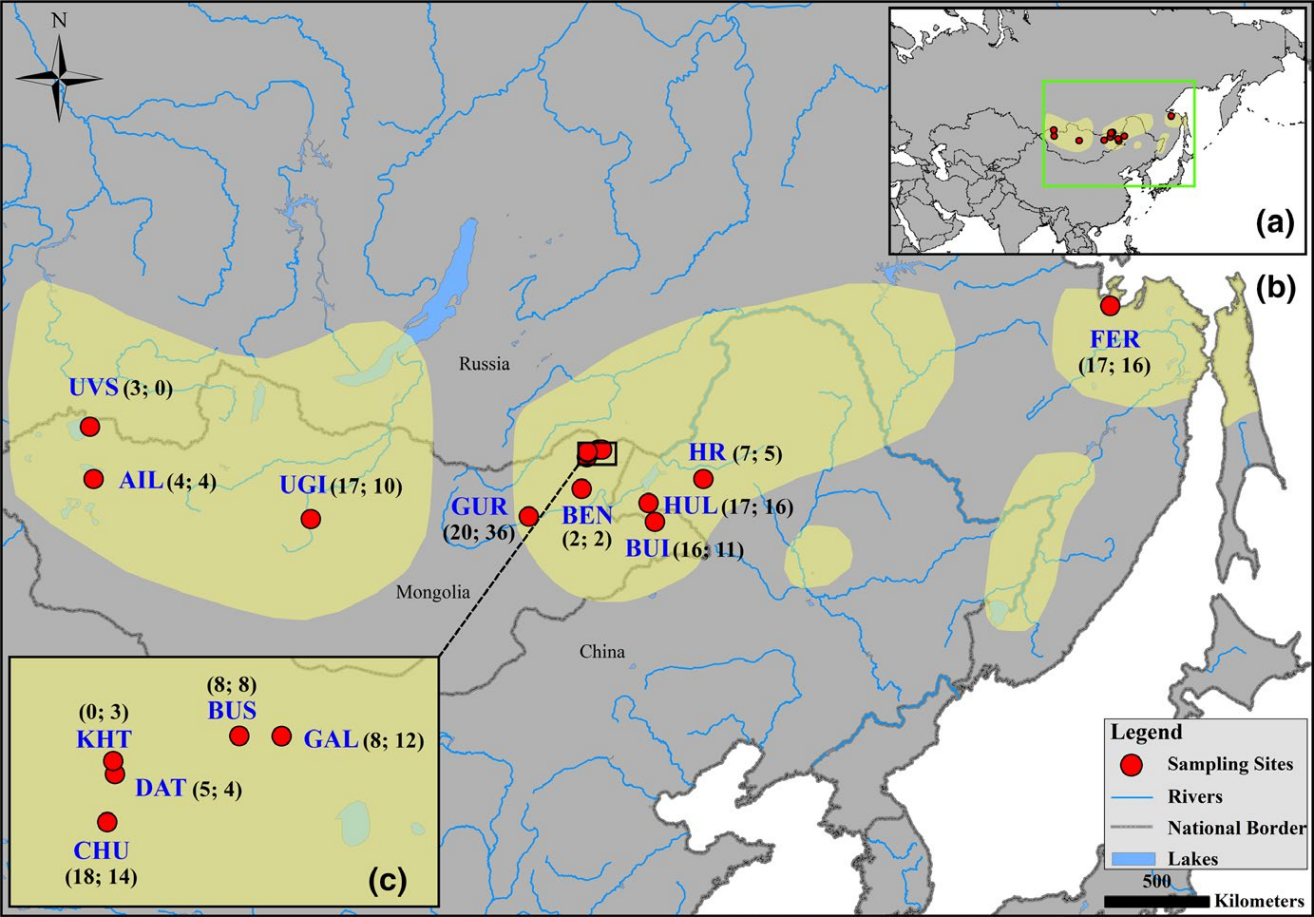
Location map of the study region for Swan Geese *Anser cygnoides* in eastern Asia (inset a), sampling sites across the breeding range (shaded pale yellow in main map b) and detail in northeast Mongolia (inset c). The blue abbreviations used for each geographical location are as follows: Uvs Lake (UVS; Mongolia), Airag Lake (AIL; Mongolia), Ugii Lake (UGI; Mongolia), Gurem Lake (GUR; Mongolia), Baruun ereen nuuriin burd (BEN; Mongolia), Chukh Lake (CHU; Mongolia), Davsan tsagaan (DAT; Mongolia), Khaichiin Tsagaan Lake (KHT; Mongolia), Bus Lake (BUS; Mongolia), Galuut Lake (GAL; Mongolia), Buir Lake (BUI; Mongolia), Hulun Lake (HUL; China), Hui River (HR; China), Ulbanskiy Bay in Far East Russia (FER; Russia). Numbers inside of each bracket represent sample sizes for males (before the semicolon) and females (after the semicolon). The extent of the breeding range shown here was derived from BirdLife International and Handbook of the Birds of the World (2018)

We collected 284 contour feather samples for Swan Geese from 14 locations within the breeding/molting areas during July to August between 2012 and 2017 (Figure 2). We rounded up flightless geese using boats, gently pushed them into corrals and nets on land. Feathers were plucked from captured geese with the feather root intact except for those from Ulbanskiy Bay in Far East Russia (FER; see Figure 2) which were collected as shed feathers by necessity from the ground just after the departure of the molting geese. Figure 2 shows the final sample sizes for each site following post hoc analysis to ensure we did not duplicate individuals in the analyses. Samples were stored in paper envelopes until laboratory analysis. Goose captures in Mongolia were carried out under licenses from the Ministry of Nature, Environment and Tourism of Mongolia (Nos. 06/2008 and 06/2862) and elsewhere in accordance with the guidance and permission (No. rcees‐ddll‐001) of Research Center for Eco‐Environmental Sciences, Chinese Academy of Sciences.

### 2.2 Laboratory procedures

Genomic DNA was extracted using the DNeasy Blood and Tissue Kit (QIAGEN) following the manufacturer's protocol with the modification of adding 20 μl 1M dithiothreitol before incubation. A singleplex fluorescent PCR targeting the avian *spindlin* gene was performed to determine the sex of each sample using the sex marker Z43B (Dawson, Dos Remedios, & Horsburgh, 2016). Alleles were separated using capillary electrophoresis on a 3730XL Genetic Analyzer with the internal size marker GS‐500LIZ ROX (Applied Biosystems). Allele size was checked and scored in the program GeneMarker HID from SoftGenetics, Inc.

Initially, three individuals were screened at 57 loci developed from domestic Swan Geese (Li et al., 2013). Subsequently, 30 loci with clear PCR product bands *via* electrophoresis were selected for further screening. A pooled DNA sample for three individuals from each breeding location was treated as the template for a 3‐primer PCR system (Schuelke, 2000) to amplify the selected 30 loci. Finally, 17 loci with high polymorphisms were chosen and allocated into five sets of multiplexing PCR (see Table S1; Set 1: ZAAS050, ZAAS004, ZAAS144; Set 2: ZAAS150, ZAAS113, ZAAS036, ZAAS182; Set 3: ZAAS023, ZAAS154, ZAAS152, ZAAS134; Set 4: ZAAS146, ZAAS020, ZAAS177, ZAAS079; Set 5: ZAAS151, ZAAS169) with forward primers fluorescently labeled. The 5 μl PCR mix contained 30 ng DNA, 2.5 μl of QIAGEN Multiplex PCR Master Mix (QIAGEN), 0.5 μl of RNase‐free water, and 0.5 μl of each primer set. The thermal conditions maintained for amplification were as follows: 95°C for 15 min, 35 cycles of 94°C for 30 s, 57°C for 90 s, 72°C for 90 s, and a final extension step of 72°C for 10 min. We separated and scored alleles using the same method described for sex identification. For quality control, 10% of the samples were re‐amplified and genotyped for all primer sets including the sex marker Z43B.

### 2.3 Individual identification

To avoid possible pseudo‐replications (i.e., feathers from the same individual), we carried out individual identification for all the 284 samples. Firstly, we assessed the markers’ power to discriminate individuals with genotype accumulation curves in “poppr” R package (Kamvar, Tab ima, & Grünwald, 2014). Program CERVUS 3.0.7 (Kalinowski, Taper, & Marshall, 2007; Marshall, Slate, Kruuk, & Pemberton, 1998) was then applied to individual identification based on the threshold obtained from genotype accumulation curves, allowing fuzzy matching with up to two mismatching loci (Pérez‐Alvarez et al., 2015). Finally, we combined information from sex identification to confirm the reliability of any potential sample pairs originating from the same individuals.

### 2.4 Standard genetic analysis

The recorded microsatellite genotypes were examined to evaluate genotyping errors, estimating potential allele dropout, and null allele frequency for each locus using MicroChecker 2.2.3 (Van Oosterhout, Hutchinson, Wills, & Shipley, 2004). Possible deviations from the Hardy–Weinberg equilibrium (HWE) and linkage disequilibrium (LD) between all locus pairs were analyzed in GENEPOP 4.0 (Raymond, 1995). Significance criteria were adjusted for the number of simultaneous tests using Sequential Bonferroni corrections (Carvajal‐Rodríguez, 2017; Rice, 1989).

The program GenAlEx 6.5 (Peakall & Smouse, 2005, 2012) was used to calculate the unbiased expected and observed heterozygosity (*uH_E_* and *H_O_*, respectively) for each locus. We estimated genetic diversity using Nei's unbiased expected heterozygosity (Nei, 1978) as this is unbiased by sample size and does not appear to be seriously affected by null alleles (Chapuis et al., 2008; Maebe et al., 2013). The allelic richness (*A_R_*) corrected for sample size was calculated with FSTAT 2.9.3 (Goudet, 2001).

### 2.5 Individual genotype‐based analysis

Given the widespread geographical dispersal of migratory birds throughout the annual cycle, the application of individual‐based assignment tests, which do not assume any predefined population boundaries (Manel, Gaggiotti, & Waples, 2005; Qi, Yang, Lu, & Fu, 2013) or population equilibrium, was considered the most appropriate analytical approach for this study. We also applied several other tests, including spatial autocorrelation analysis (SAA), assignment index correlation, and first‐generation immigrant (FGM) detection.

**FIGURE 3:**
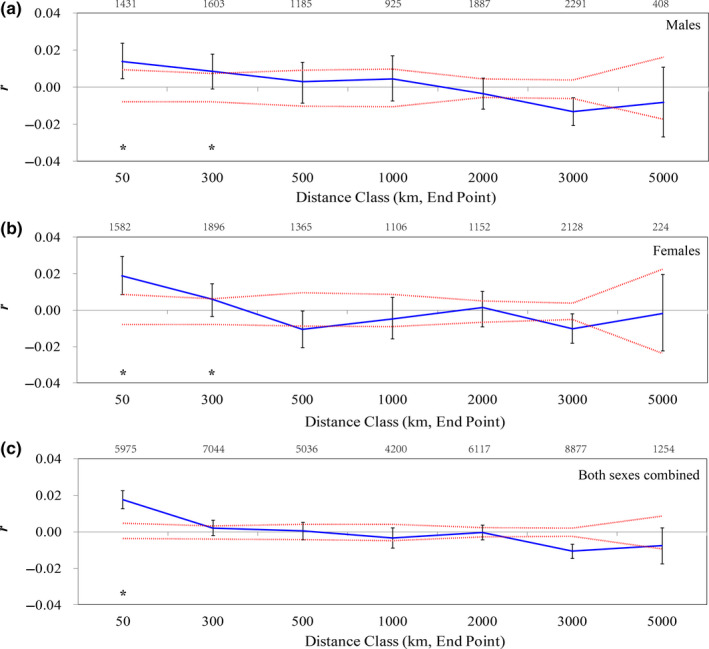
Results of the spatial autocorrelation analysis of Swan Geese *Anser cygnoides* based on all sampled individuals. Correlograms of the autocorrelation coefficient (*r*, the blue solid lines) for (a) males (*n* = 140), (b) females (*n* = 138), and (c) both sexes combined (*n* = 278) were plotted for six gradual increasing geographical distance classes. Sample sizes for each distance class are presented above each panel. The red dotted lines represent the 95% upper and lower confidence intervals of *r*. Significant spatial structure is marked with asterisks when *r* exceeds the null distribution and the error bars do not overlap zero

SAAs were performed for all sampled individuals (*n* = 278) as well as for each sex separately (140 for males and 138 for females) with GenAlEx 6.5 (Peakall & Smouse, 2005, 2012) to provide a multivariate and multilocus evaluation on the spatial genetic structure. This technique calculates an autocorrelation coefficient (*r*) for individuals collected within the bounds of predefined distance classes. Under a model of restricted dispersal, genetic and geographical distances will be positively correlated over short distances. In order to guarantee enough statistical power and avoid noise in the confidence limits that can be caused by a biased sample size, each distance class must contain enough pairwise comparisons (Peakall & Smouse, 2005). To meet this assumption, we defined the final distance classes in our study as follows: (a) 0–50 km, (b) 51–300 km, (c) 301–500 km, (d) 501–1,000 km, (e) 1,001–2,000 km, (f) 2,001–3,000 km, and (g) 3,001–5,000 km (Figure 3). Values of *r* above the upper 95% confidence interval indicate a significantly positive genetic structure. The first distance class where *r* is no longer significant can be used to indicate the extent of detectable structure (Blackmore & Heinsohn, 2015; Peakall, Ruibal, & Lindenmayer, 2003). We tested for significance using 9,999 random permutations, and 95% confidence intervals for estimates of *r* were determined by 9,999 bootstraps.

**FIGURE 4:**
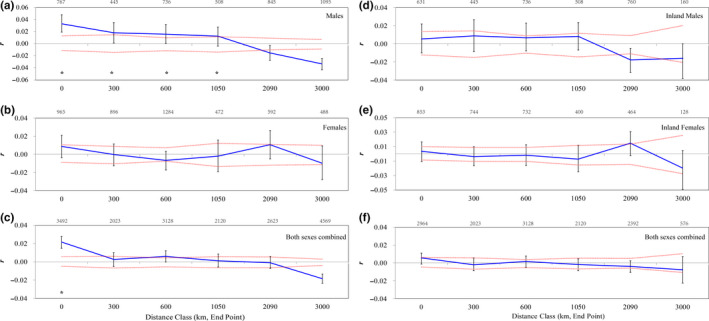
Spatial autocorrelograms for Swan Geese *Anser cygnoides* based on localities that are approximately equidistant (AIL, UGI, GUR, CHU, HUL, and HR; see Figure 2 for details) including (a‐c) or excluding (d‐f) the coastal group (FER). The autocorrelation coefficient (*r*, the blue solid lines) for (a, d) males (*n* = 98 and 81, respectively), (b, e) females (*n* = 98 and 82, respectively), and (c, f) both sexes combined dataset (*n* = 196 and 163, respectively) is plotted for six gradual increasing geographical distance classes. Sample sizes for each distance class are presented above each panel. The red dotted lines represent the 95% upper and lower confidence intervals of *r*. Significant spatial structure is marked with asterisks when *r* exceeds the null distribution and the error bars do not overlap zero

Since most of our sampling localities were spatially aggregated in eastern Mongolia, and the SAA results might be biased from the overrepresentation of short distance, the same analyses were then performed for six localities (AIL, UGI, GUR, CHU, HUL, and FER) which are approximately equidistant for obtaining realistic results. We also conducted SAAs including/excluding the coastal locality (FER) to detect any dissimilarity in the local genetic structure at the whole region versus the inland region (Figure 4).

To obtain information about current dispersal between localities, we carried out an assignment analysis and looked for FGM with the program GENECLASS 2.0 (Cornuet, Piry, Luikart, Estoup, & Solignac, 1999; Ruan et al., 2018). The Bayesian method was chosen since it has been described as best adapted in assigning/excluding individuals to locations (Rannala & Mountain, 1997). For both assignment analysis and FGM detection, we used 10,000 replicates, setting the alpha level for MCMC simulations (Paetkau, Slade, Burden, & Estoup, 2004) at 0.01 and for the assignment threshold at 0.05 (Ceresa, Belda, Kvist, Rguibi‐Idrissi, & Monrós, 2015). Analyses were performed for males and females separately to avoid bias that could arise due to unequal representation of the sexes as recommended by Salgueiro, Palmeirim, Ruedi, and Coelho (2008). For FGM detection, we used the *Lh*, which described the likelihood of finding a given individual in the population in which it was sampled. This statistical criterion is convenient whenever not all potential source sites were sampled for the study species (Paetkau et al., 2004; Qi et al., 2013). Individuals were excluded from their sampling localities if the Bayesian probability was less than 0.05, or assigned to that locality if the Bayesian probability was equal to or greater than 0.05 (Ginson, Walter, Mandrak, Beneteau, & Heath, 2015). Individuals that could be excluded from all locations on this basis were considered to be from an unsampled location (Pruett, Li, & Winker, 2018).

Mean assignment index correlation (*mAIc*; Favre, Balloux, Goudet, & Perrin, 1997) was also used to detect SBD with the program GenAlEx 6.5 (Peakall & Smouse, 2005, 2012). Negative *mAIc* values suggest higher frequency of rare genotypes than expected, which indicates high frequency of dispersal (Qi et al., 2013). The advantage of this method includes allowing each geographical group of samples to be tested independently, which can thus provide dispersal information at different geographical scales. Male and female values were calculated for sampling locations with total sample size equal or greater than 20 (CEN, CEP, CHU, GUR, BUI, HUL, FER). In a species without SBD, we expected that *mAIc* would not differ between sexes. Statistical significance was assessed using Mann–Whitney *U* tests implemented in GenAlEx 6.5 (Peakall & Smouse, 2012).

### 2.6 Allele frequency‐based analysis

Isolation‐by‐distance (IBD) analyses were performed for each sex and subsequently for the whole dataset. A pairwise genotype distance was computed against geographical distance matrix (natural logarithm transformed) with the program GenAlEx 6.5 (Peakall & Smouse, 2005, 2012). Statistical significance was tested by random permutation (999 permutations) against a null hypothesis of no relationship between genetic and geographical distance. Only locations contributing 20 or more individuals were used in this part of the analysis.

Separate AMOVAs were performed with the program GenAlEx 6.5 (Peakall & Smouse, 2005, 2012) for each sex and for the combined dataset including males and females. Males and females were assigned to “regions” in the terminology used here, with sampling locations of the respective relevant sex again included as “populations”, for elucidating whether the amount of within‐ and between‐region genetic variation was similar for males and females. This hierarchical analysis allowed a simultaneous comparison of the differentiation among sexes and locations. Under strong SBD, we would expect significant differentiation between the two sexes. Randomization tests (1,000 permutations) were performed to test significant departure from the null hypothesis of no genetic differentiation. Only locations contributing 20 or more individuals were used in this part of analyses.

Five other statistical descriptors, including average expected heterozygosity within a site (gene diversity; *H_S_*), mean relatedness (*R*), the variance of *AIc* (*vAIc*) *vAIc*, and two *F*‐statistic parameters (*F_IS_*, a measure of the within‐population heterozygote deficit; *F_ST_*, a measure of the among‐population heterozygote deficit) among individuals were calculated separately for males and females with FSTAT 2.9.3 (Goudet, 1995). Statistical significance for all these indices was determined by 10,000 randomizations. A two‐sided permutation was used to test for significant differences (Qi et al., 2013). Only locations contributing 20 or more individuals were included in the analyses.

## 3 RESULTS

### 3.1 Individual identification

**FIGURE 5:**
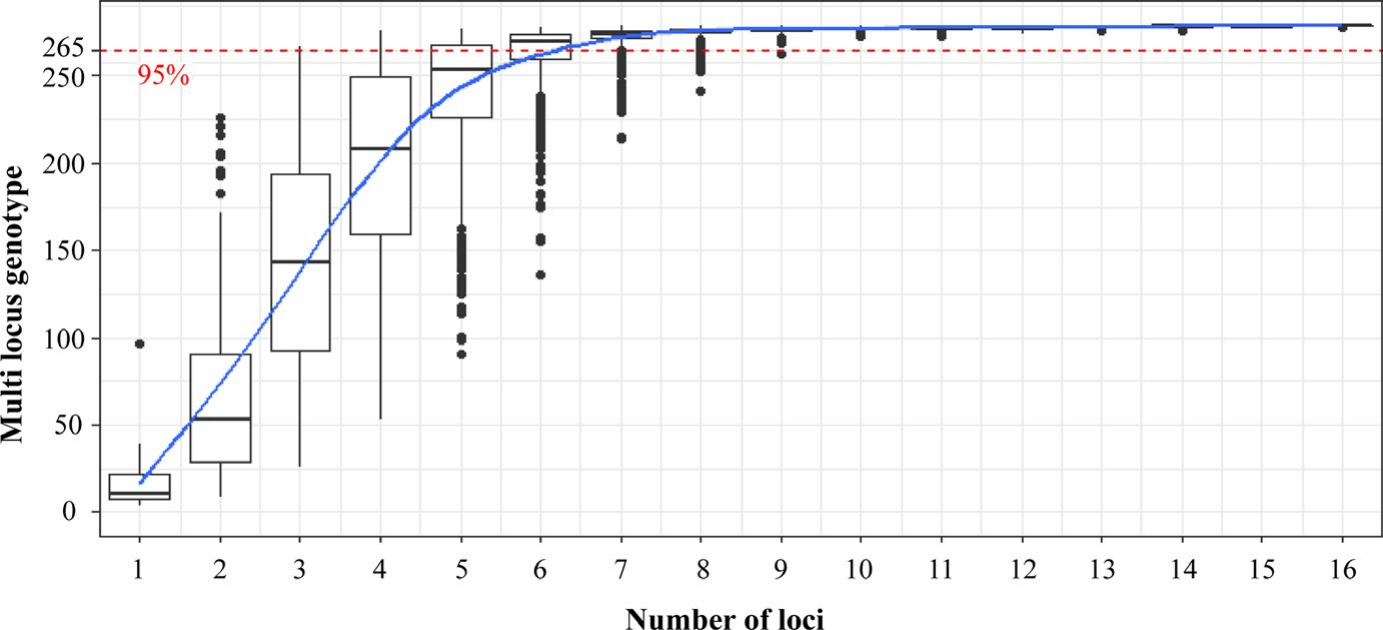
Genotype accumulation curve for Swan Geese *Anser cygnoides* based on 17 microsatellite markers. Box plots were constructed by randomly sampling loci 1,000 times. The 95% of the number of multilocus genotypes discriminated is indicated by a dashed line

A total of 284 feather samples from 14 sampling locations were genotyped at 17 polymorphic microsatellite loci. The dataset of 17 loci was sufficiently powerful to discriminate 95% of the multilocus genotypes with at least seven loci and unique individuals with ten loci (Figure 5). Twelve pairs of potential replicates from FER matching at a minimum of ten loci but mismatching at up to two loci were obtained. After considering discrepancy regarding sex identification, we discarded six samples from FER and were able to create a list of 278 unique individuals for 14 sampling sites including 140 males and 138 females (Figure 2). However, seven of the sites had sample sizes less than 20 and were excluded from genetic diversity and population level analysis due to potential deviation caused by sampling bias.

### 3.2 Standard genetic diversity

The *H_O_* within loci across the sampled groups ranged from 0.061 to 0.889 and *uH_E_* ranged from 0.036 to 0.888 (Table S1). The average allele richness (*A_R_*) ranged from 4.353 in GAL to 5.050 in BUI, while the highest and lowest average *uH_E_* was detected in BUI (0.545) and UGI (0.473), respectively. Deviations from HWE (*p* < .00042) were observed at multiple loci and sampling locations (ZAAS036—GUR; ZAAS134—CHU, GAL, and GUR; ZAAS151—CHU), and there was evidence of linkage disequilibrium (*p* < .00005) between ZAAS036 and ZAAS134. Null alleles appeared in more than one location for both ZAAS036 (CHU, GUR, HUL), ZAAS134 (UGI, CHU, GUR), and ZAAS151 (UGI, BUI, HUL). Therefore, all these three loci were removed, and finally, 14 loci were used for subsequent SBD analyses.

### 3.3 Individual‐based analysis

According to the outcomes of SAAs for all the samples (Figure 3), significant positive spatial autocorrelation among genotypes was identified within distance classes 0–50 km for males (*n* = 140; *r* = .014, 95% CI 0.009, −0.008), females (*n* = 138; *r* = .019, 95% CI 0.009, −0.009), and the combined dataset (sexes combined, *n* = 278; *r* = .018, 95% CI 0.005, 0.004). Values of *r* decreased with increasing geographical distance in all datasets and in the second distance class for all three datasets. The overall shape of the correlogram was similar for males and females. The *x*‐intercept for females (372.481 km) was much smaller than for males (1,548.769 km).

When the SAAs were restricted to the six localities that were approximately equidistant, patterns were different. Both females (*n* = 98; *r* = .009, 95% CI 0.011, −0.009) and the combined dataset (*n* = 196; *r* = .022, 95% CI 0.005, −0.005) revealed a positive and significant *r* value at only the 0 km distance class (*p* = .049 and 0.001 respectively; Figure 4). When the coastal locality was excluded, none of the three datasets (inland males, inland females, inland whole dataset; Figure 4) revealed significant spatial autocorrelation at any distance class.

Out of a total 140 samples, GENECLASS identified 10 males to be FGM and 130 males to be residents. Among females, nine were regarded as FGM out of the total 138 individuals (Table S2). Results from the assignment‐exclusion test assigned most of the potential first‐generation immigrants (12/18) to more than one locality with similar probabilities (Table S2). Another male (AIL7) seems to come from unknown location since it has been excluded from all our existing localities according to its assignment probability value. Meanwhile, five other geese (three males: BUI30, FER4, FER6; two females: GUR26, HUL2; Table S2) exhibited extremely low probability to only one site. However, these samples may represent individuals who cannot be accurately assigned due to a lack of information in the data, the low or similar assignment probabilities could also be indicative of admixed ancestry (Bergl & Vigilant, 2007). We thus come to the conservative conclusion that we failed to detect any FGM.

**FIGURE 6:**
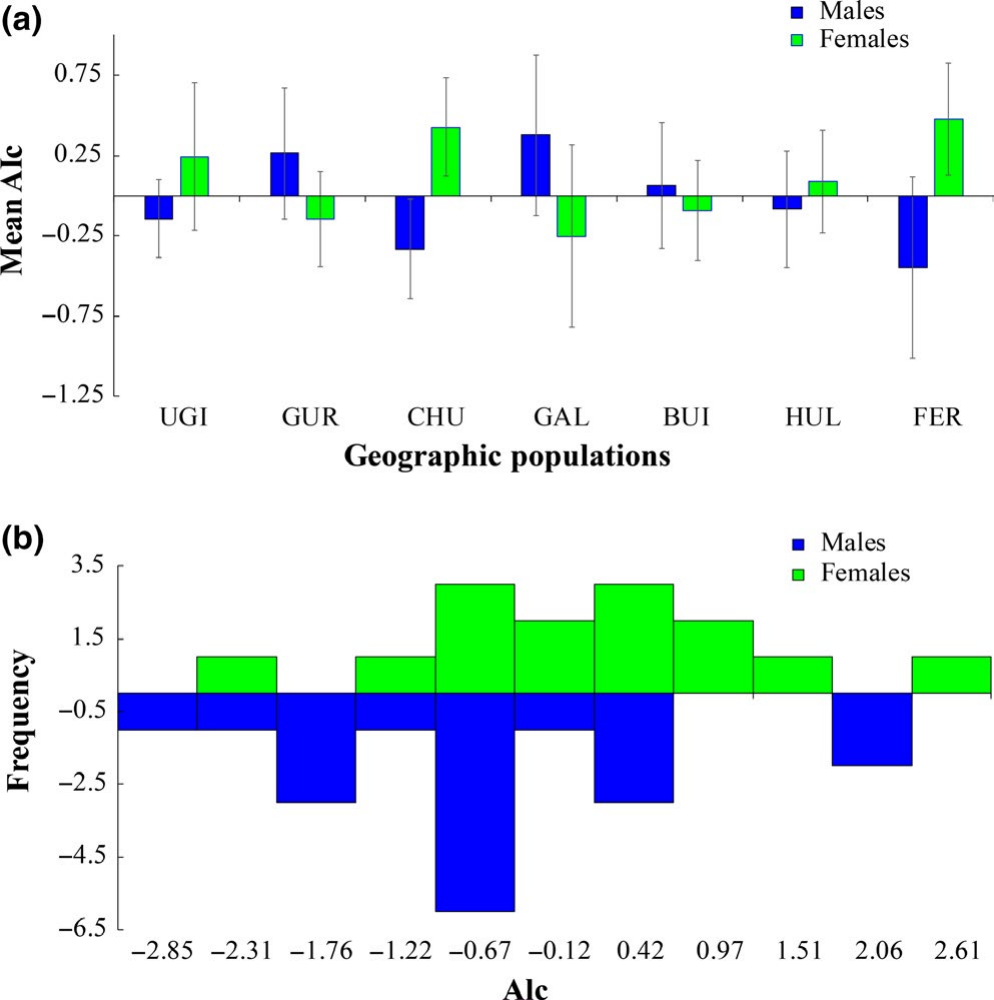
(a) Comparisons of mean assignment index correlation (*mAIc*) values between males and female Swan Geese *Anser cygnoides* from the seven sampling locations with total sample size equal or greater than 20; (b) detailed AIc distribution of the inland group (all locations without FER) for males and females. Abbreviations used for each of the sampling locations are explained in Figure 2

The *mAIc* values were negative for males in four locations (UGI, CHU, HUL, FER; Figure 6) and positive in the others, suggesting that the rare genotypes were more frequent in males for these locations. In CHU, the difference in *mAIc* values between males (*n* = 18) and females (*n* = 14) were marginally nonsignificant (*Z* = 1.862, *p* = .063), while not significant (*p* > .05) for all the other locations. Moreover, the *mAIc* value was negative for males (−0.077) and positive for either females (0.075) or the combined dataset when considering all the seven sampling groups (UGI, GUR, CHU, GAL, BUI, HUL, FER), but the difference still failed to attain statistical significance (*Z* = −0.443, *p* = .658). A similar pattern appeared when pooling data including both sexes from the inland group (*Z* = 0.211, *p* = .833).

### 3.4 Allele frequency‐based analysis

Mantel tests revealed a significant and positive relationship between genetic and geographical distance for the combined dataset, as well as the males (*p_IBD_* = .001 for both, Table 1), which suggest a scenario of IBD pattern in both datasets. When the analysis was restricted only to those approximately equidistant localities, a similar pattern emerged for the dataset which included the coastal locality (*p* = .002 and .022 for males and combined dataset, respectively). In contrast, this test was nonsignificant when restricted to the inland group (*p* > .05).

**TABLE 1:** Detection of genetic differentiation (*F_ST_*) and isolation by distance (IBD) of Swan Geese *Anser cygnoides* for males, females, and the whole dataset. *df*, degree of freedom; *p_AMOVA_*, *p*‐value for the analysis of molecular variance (AMOVA); *R_xy_*, correlation coefficient of IBD; *p_IBD_*, *p*‐value for the analysis of IBD

Testing group	Location number	*F_ST_*	Variation among localities	*df*	*p*_AMOVA_	*R_xy_*	*p*_IBD_
Males	7	0.019	2%	6	.001	.075	.001
Females	7	0.022	2%	6	.001	.020	.240
Whole dataset	14	0.018	2%	12	.001	.044	.001

The overall genetic differentiation level among seven geographical locations was low but significant for both sexes (*F_ST_* = 0.019, 0.022 for males and females respectively; *p_AMOVA_* = .001; Table 1), which was consistent with the analysis of the entire dataset (*F_ST_* = 0.018; *p_AMOVA_* = .001). The variation among locations was extremely low (2%) for both sexes. Interestingly, this variation component became 0% when considering the two sexes as two distinctive regions. All estimated population genetic descriptors are presented in Table 2. The more dispersing sex should have higher *H_S_, F_IS_* and *vAIc*, a lower *F_ST_* and *R*. The general trend of our result was more indicative of MBD (lower *F_ST_* and *R*, higher *H_S_* and *vAIc* in males), but none of them showed statistically significant differences between males and females (*p* > .05). Therefore, our results did not find clear support for SBD in Swan Geese at the landscape level.

**TABLE 2:** Differences between males and females of Swan Geese *Anser cygnoides* in gene diversity (*H_S_*), measurement of the within‐population heterozygote deficit (*F*_IS_), measurement of the among‐population heterozygote deficit (*F*_ST_), mean relatedness (*R*), and variance of the *AIc* value (*vAIc*). *N*, sample size; *p*, *p*‐value for the corresponding test

Sex	*N*	Tests of sex‐biased dispersal				*vAIc*
		*H_S_*	*F_IS_*	*F_ST_*	*R*	
Male	140	0.4680	0.0316	0.0213	0.0405	13.6443
Female	138	0.4641	0.0325	0.0223	0.0422	12.1188
*p*		.7328	.9729	.9096	.9124	.6098

## 4 DISCUSSION

Our main study objective was to investigate the genetic signal of SBD in the Swan Goose by means of examining 14 biparentally inherited genetic markers among birds caught across the full expanse of their breeding range. The results were generally consistent with our prediction that the species would not show conspicuous SBD. Firstly, although the differentiation level among sampling localities was low, dispersal was relatively limited. Secondly, we found no evidence of sex‐specific dispersal patterns in Swan Goose at landscape level in a northern hemisphere low latitude breeding goose species for which we currently lack behavioral observations on dispersal. Individual‐based and allele frequency‐based analyses jointly showed a lack of marked MBD in this species, typical of other Anatidae species at the landscape level. SAA results displayed distinct local genetic structures within the dataset, when considered with and without the coastal group (FER, See Figure 2), which intriguingly suggests some local female‐biased dispersal in the eastern section of our study area.

### 4.1 Genetic diversity and differentiation

The low genetic differentiation (*F_ST_* = 0.018, Table 1) in our study of Swan Geese is similar to that previously detected in other geese species, including the Bean Goose *Anser fabalis* (Honka et al., 2017), Greater White‐fronted Goose *Anser albifrons* (Ely et al., 2017), and Greylag Goose (Pellegrino et al., 2015). The nuclear diversity in all sampling localities, measured as observed heterozygosity, was low (0.438–0.512) compared to the Greater White‐fronted Goose (Ely et al., 2017), but similar to that in the Lesser White‐fronted Goose (0.51) which has shown recent strongly declining population size (Ruokonen, Andersson, & Tegelström, 2007). All the genetic parameters we analyzed were similar across the distribution range for seven sampling localities with sample size greater than 20 (Table S1).

### 4.2 Range‐wide genetic structure

Interpretation of the level of spatial genetic structure in the Swan Goose seems to be dependent on sampling density in eastern Mongolia. A similar pattern of fine‐scale genetic structure was found in SAAs in both sexes, as well as for both sexes combined (Figure 3). When considering all the samples, both males and females showed significantly positive genetic structure within the shortest distance class, but this gradually diminished with increasing distance. However, restricted to sampling localities approximately equidistant apart suggested a significantly positive genetic structure for males, which was absent in females (Figure 4).

Unlike a genetic study of Canada/Cackling Geese *Branta canadensis/Branta hutchinsii*, which provided evidence for MBD (Leafloor, Moore, & Scribner, 2013), we recovered a similar pattern of fine‐scale genetic structure in both sexes for Swan Geese within colonies (0 km, Figure 4, panel a–c). The philopatry for both sexes originating from cultural transmission (Harrison et al., 2010) is likely one of the reasons responsible for this pattern. Female natal fidelity is generally assumed to be the case for most geese, yet male philopatry has been documented for the Lesser Snow Goose *Chen caerulescens* (Cooke, 1978), Canada Goose (MacInnes, 1966), and Brant *Branta bernicla* (Abraham, Ankney, & Boyd, 1983). Other studies have demonstrated the high degree of adult winter site fidelity in the Greenland White‐fronted Goose *Anser albifrons flavirostris* (Weegman et al., 2015), Pink‐footed Goose *Anser brachyrhynchus* (Fox et al., 1994), and Light‐bellied Brant *Branta bernicla hrota* (Harrison et al., 2010). Many goose species exhibit complex social organization arising from extended parental care which not only instills the use of traditional breeding–staging–wintering areas (Ely et al., 2017), but also enables offspring to learn species‐specific characteristics related to mate choice and preferences from parents (Harrison et al., 2010). Ultimately, the maintenance of local population structure in goose species could be further facilitated by site fidelity of both sexes, as well as the integrative effect of behavioral‐related factors (e.g. extended parental care and its associated cultural transmission) across the generations in Swan Geese.

The stronger signs of IBD among males than that among females indicate that males exhibit more limited dispersal across sampling locations. Thus, males may primarily drive the landscape level genetic structure of the Swan Goose (Table 1). Although the statistical power of this test was relatively low (*R_xy_* = .075), we also detected intersexual difference in SAAs (Figure 4, panels a and b). A positive genetic signal for males at the 1,050 km distance class (not found for females) implies that female Swan Geese tend to disperse further than males at the local scale, resulting in males living in adjacent groups being, on average, more genetically related with each other than with more distant individuals. These findings support genetic evidence of local female‐biased dispersal for the Swan Goose.

In contrast to the IBD pattern that we found in males, the absence of genetic structure among females belonging to different localities is suggestive of a random spatial distribution of genotypes within the study area. This result was unexpected since previous studies on geese proposed the males as the primary vector of dispersal and genetic mixing at large spatial scales (Jeugd, 2001; Lecomte, Gauthier, Giroux, Milot, & Bernatchez, 2009; Lessells, 1985; Ruokonen et al., 2010). Female philopatry (natal site fidelity) in goose species is often invoked as a predictor of population structure (Pearce, McCracken, Christensen, & Zhuravlev, 2009), as well as a behavioral equivalent of isolation by distance (Greenwood, 1980; Pearce et al., 2009). However, our result is inconsistent with this assumption, which confirms that females could also play a role in gene exchange among localities.

We suggest that the intersexual differences in genetic structure at other distance classes beyond the first one are likely reflect the intersexual dissimilarity in natal dispersal distance. Female Swan Geese are solely responsible for nest construction (Kear, 2005), so are likely to exhibit a stronger tendency to disperse from their natal group if nesting sites were limiting in any way. This may be the case for the Swan Goose, because the core breeding area (the transboundary Dauria region, Fox & Leafloor, 2018) experiences 25‐ to 35‐year cyclical patterns of precipitation. The recent drought phase has affected local abundance, extent, and quality of wetlands in this region and hence the local availability of breeding habitat for the species (Fox & Leafloor, 2018). At larger geographical scales, lake loss and shrinkage of open water areas throughout the Mongolian Plateau during the last two decades (John et al., 2016; Tao et al., 2015) associated with anthropogenic effects (e.g. habitat modification, illegal hunting, and large‐scale raising of grazing livestock) may further reduce habitat quality and availability here for the Swan Goose. Even if, at their first lifetime breeding attempt, females tend to return to their natal site or adjacent area for breeding, the reduction in nesting site availability in familiar areas likely force first‐time breeding females to seek suitable nesting sites elsewhere, leading to more random spatial distribution of female genotypes than that of males at the local scale.

Moreover, our results provided some evidence for the scale dependence of dispersal in the Swan Goose. Genetic structure at local scale disappeared after the exclusion of the coastal locality for all the three inland datasets (Figure 4, panels d–f), suggesting there may be two contrasting dispersal patterns for the same population at different spatial scales. This kind of scale‐dependent dispersal pattern has also been observed in other species (Li et al., 2019; Vangestel, Callens, Vandomme, & Lens, 2013) and could be partly explained by variation in landscape configuration and the density‐dependent factors such as current individual densities (Mora, Map elli, Gaggiotti, Kittlein, & Lessa, 2010; Morton et al., 2018) at different spatial scales. For Swan Geese, the former continuous breeding area has become highly fragmented (Fox & Leafloor, 2018; See Figure 1). Currently, the population size for the isolated coastal group was estimated to be only a few hundreds, while all the other 55,000–65,000 birds are inhabit inland area (Fox & Leafloor, 2018). Besides the vast difference in population size, the suitable habitats for coastal geese also seem to be scarcer when compared with the inland group. Located at the eastern limit of the breeding distribution, the coastal group has a more restricted and patchier distribution (Lake Udyl, Schastye Bay, and northern Sakhalin Island), while the inland group exploits discontinuous, but widespread habitats associated with numerous lakes distributed across the Mongolian Plateau. The increasing divergence of the coastal group from the inland is therefore associated with scale‐dependent distribution of patchy breeding resources, which would ultimately lead to the heterogeneous patch occupancy and differential individual turnover within patch networks.

All our feather samples were collected during the flightless molting period (confirmed by phenology data from a tracking study (Batbayar et al., 2011). The core breeding/molting area (the transboundary Dauria region, Fox & Leafloor, 2018) is known to gather nonbreeders from all of the known global summering areas including the Amur River basin in the vicinity of the FER locality (Goroshko, 2004), as well as local breeding birds. Feathers were plucked from molting adults in Mongolia but were collected post molt from Swan Geese of unknown provenance in FER. We should therefore be extremely prudent about concluding too much from results from FER feathers. Although there was an extremely low probability for us to have sampled geese in FER which had migrated from elsewhere, we cannot rule out the possibility that goose feathers sampled in FER were from geese that were migrating through, rather than breeding there.

### 4.3 Why no SBD at the landscape level?

Several nonexclusive explanations could contribute to the apparent absence of SBD at the landscape level for the Swan Goose in our study. Firstly, the monogamous and “mate defense” mating system of Swan Geese is less prone to the evolution of sex‐specific strategies. Intersexual asymmetries in limiting resources is generally assumed to be one of the hypotheses explaining sex bias in dispersal for socially monogamous avian species with a “resource defense” mating system (Greenwood, 1980; Lawson Handley & Perrin, 2007). In those species, males benefit more from site fidelity than females who need to search for mates and sites for reproduction (Liebgold, Gerlach, & Ketterson, 2013). However, for genetic and long‐term monogamous species such as geese (Toft & Wright, 2015), the typical “mate defense” mating system (Mabry et al., 2013) means that the distribution of females is not primarily determined by the resources that are held by males (Clarke et al., 1997). Therefore, it is unlikely that limiting resources in some way asymmetrically affect the sexes in the Swan Goose, leaving little opportunity for SBD to evolve.

Secondly, the severe fragmentation of Swan Goose breeding habitats in the last 25 years (Fox & Leafloor, 2018) may have constrained any long‐distance dispersal behavior. Previous findings suggest that the extent of differential gene flow between sexes may be scale‐dependent (Vangestel et al., 2013). While local scale dispersal (which we described above) often involves movements within continuous patches of suitable habitat (Liebgold et al., 2013; Vangestel et al., 2013), long‐distance movement implies seeking remaining habitat patches that are widely separated by inhospitable terrain (Tittler, Villard, & Fahrig, 2009; Wesołowski, 2015; Woltmann, Sherry, & Kreiser, 2012). Since such long‐distance movements through unknown and potentially hostile environments are bound to bear both mortality risk (Johnson, Fryxell, Thompson, & Baker, 2009) and energetic cost (Bonte et al., 2012), the prevalence of dispersal must be maintained by selective forces (Perrin & Mazalov, 1999). Such selective forces are likely to be similar for both sexes, a factor which will ultimately balance the dispersal rates of females and males at a regional scale (Yannic, Basset, Büchi, Hausser, & Broquet, 2012).

Thirdly, lack of power may have prevented us from identifying asymmetrical sex dispersal patterns at the landscape level. On the one hand, it has been demonstrated that the statistical power of autocorrelation analysis to detect sex‐dependent dispersal is highest at the spatial scale where the level of aggregation of relatives is highest (Banks & Peakall, 2012; Liebgold et al., 2013). However, to date, we know little about either the natal dispersal or family group structure in Swan Geese, which requires further fieldwork tracking individually marked birds in the future. We therefore lack spatial resolution for the landscape level analyses. Although we used 50 km as such a criteria for Swan Geese (Zhu et al., 2020), in some related species (such as the Greylag Goose) breeders recruit within 30 km of their nesting site (Nilsson & Persson, 2001). In contrast, 38% of tracked Barnacle Geese show a natal dispersal distance of less than 100 km (van der Jeugd, 2013). It is clear that we need individually marked Swan Geese of known age to understand better natal dispersal rates and the social structure of the Swan Goose in the immediate future.

On the other hand, we cannot completely reject the hypothesis that the effects of large‐scale molt migration may have hampered our ability to detect any clear SBD patterns for the Swan Goose. Failed breeders and nonbreeding Swan Geese are known to undertake molt migration, potentially to wetlands remote from their breeding areas like other temperature or sub‐Arctic‐nesting geese such as the Greylag Goose (Nilsson, Kahlert, & Persson, 2001), Lesser White‐fronted Goose (Aarvak & Øien, 2003), and Bean Goose (Nilsson, de Jong, Heinicke, & Sjöberg, 2009). Therefore, our sampling strategy was likely potentially biased toward molting individuals rather than breeding geese (although these are extremely difficult to catch without causing disturbance). For this reason, our results may not be representative of the population as a whole, especially because more mobile nonbreeders made up a high percentage of our samples from Swan Geese and those of other studies (Goroshko, 2004). This may be the case here, because we detected marginally nonsignificant MBD for the CHU locality (significant difference between males and females, see Figure 6), and more than half of our sampled populations showed negative *mAIc* values for males. Therefore, we cannot rule out the possibility of weak MBD in Swan Goose, which our study was not able to detect with certainty. A previous study highlighted the fact that the fine‐scale avian genetic structure may differ depending on the stage of the breeding cycle when birds were sampled (Lecomte et al., 2009). For this reason, it would be highly advantageous to mount additional sampling efforts, which focus on taking samples from individuals at brood rearing sites or at other breeding stages, especially in FER, as well as amassing sequential field observations of neck collared individuals to further confirm the lack of SBD in this threatened species.

## CONFLICT OF INTEREST

We declare no conflicts of interest.

## AUTHOR CONTRIBUTION

**Qin Zhu:** Conceptualization (lead); data curation (lead); formal analysis (lead); methodology (lead); software (lead); writing – original draft (lead); writing – review & editing (lead). **Iderbat Damba:** Investigation (lead); project administration (supporting). **Qingshan Zhao:** Conceptualization (supporting); funding acquisition (lead); project administration (lead); writing – review & editing (supporting). **Kunpeng Yi:** Funding acquisition (lead); investigation (lead); project administration (lead); writing – review & editing (supporting). **Nyambayar Batbayar:** Investigation (lead); project administration (supporting). **Tseveenmyadag Natsagdorj:** Investigation (lead); project administration (lead). **Batmunkh Davaasuren:** Investigation (lead); project administration (lead). **Xin Wang:** Funding acquisition (lead); investigation (supporting); project administration (lead); software (supporting). **Sonia Rozenfeld:** Investigation (lead); project administration (lead); resources (supporting); writing – review & editing (supporting). **Sachiko Moriguchi:** Data curation (supporting); formal analysis (supporting); funding acquisition (supporting); resources (supporting); software (supporting); supervision (supporting); writing – review & editing (lead). **Aibin Zhan:** Conceptualization (lead); methodology (lead); supervision (supporting); writing – review & editing (lead). **Lei Cao:** Conceptualization (lead); funding acquisition (lead); investigation (lead); project administration (lead); resources (lead); supervision (lead); writing – review & editing (lead). **Anthony D. Fox:** Conceptualization (lead); investigation (lead); supervision (lead); writing – review & editing (lead).

### Open Research Badges

This article has earned an Open Data Badge for making publicly available the digitally‐shareable data necessary to reproduce the reported results. The data is available at https://doi.org/10.5061/dryad.fttdz08q0.

## Supporting information

Table S1‐S2

## Data Availability

A data package including microsatellite allele length has been deposited to Dryad data repository: https://doi.org/10.5061/dryad.fttdz08q0.
