# Supplementary material for: Lack of conspicuous sex‐biased dispersal patterns at different spatial scales in an Asian endemic goose species breeding in unpredictable steppe wetlands
Source: Ecol Evol. 2020 Jun 27;10(14):7006–20. doi: 10.1002/ece3.6382 (PMC7391341; doi:10.1002/ece3.6382)
Supplement: Supplementary file 1 — Table S1‐S2 [file ECE3-10-7006-s001.docx]

**Table S1**. Genetic parameters for Swan Geese *Anser cygnoides* from seven locations with sample size equal or greater than 20 based on 17 microsatellite loci. For abbreviations for the sampling locations, see Figure 1.

| **Loc** | **Index** | **ZAAS**  **050** | **ZAAS**  **004** | **ZAAS**  **144** | **ZAAS**  **150** | **ZAAS**  **113** | **ZAAS**  **182** | **ZAAS**  **036** | **ZAAS**  **023** | **ZAAS**  **154** | **ZAAS**  **134** | **ZAAS**  **152** | **ZAAS**  **146** | **ZAAS020** | **ZAAS**  **079** | **ZAAS**  **177** | **ZAAS**  **151** | **ZAAS**  **169** | **Ave.** | |
| --- | --- | --- | --- | --- | --- | --- | --- | --- | --- | --- | --- | --- | --- | --- | --- | --- | --- | --- | --- | --- |
| UGI | *r* | no | no | no | no | no | no | no | no | no | 0.1002 | no | no | no | no | no | 0.1016 | no | - |  |
|  | *A* | 3.000 | 8.000 | 9.000 | 3.000 | 4.000 | 2.000 | 15.000 | 2.000 | 2.000 | 7.000 | 4.000 | 4.000 | 3.000 | 4.000 | 4.000 | 5.000 | 6.000 | 5.000 |  |
|  | *A_r_* | 3.000 | 7.645 | 8.791 | 2.999 | 3.740 | 1.985 | 14.222 | 2.000 | 2.000 | 6.610 | 3.985 | 3.662 | 2.677 | 3.985 | 3.922 | 4.481 | 5.662 | 4.786 |  |
|  | *H_o_* | 0.519 | 0.778 | 0.889 | 0.444 | 0.407 | 0.111 | 0.815 | 0.333 | 0.185 | 0.519 | 0.556 | 0.222 | 0.111 | 0.741 | 0.296 | 0.370 | 0.741 | 0.473 |  |
|  | *uH_E_* | 0.561 | 0.728 | 0.820 | 0.377 | 0.381 | 0.107 | 0.921 | 0.283 | 0.230 | 0.704 | 0.610 | 0.209 | 0.108 | 0.643 | 0.381 | 0.535 | 0.734 | 0.490 |  |
|  | *P_hw_* | 0.3636 | 0.8754 | 0.5470 | 1.0000 | 0.7924 | 1.0000 | 0.0375 | 1.0000 | 0.3582 | 0.0183 | 0.3522 | 1.0000 | 1.0000 | 0.9312 | 0.1086 | 0.0119 | 0.4975 | - |  |
|  | *P_LD_* | 0.0000 | 0.0000 | 0.0000 | 0.0000 | 0.0000 | 0.0000 | 0.0011**^*^** | 0.0000 | 0.0000 | 0.0011**^*^** | 0.0000 | 0.0000 | 0.0000 | 0.0000 | 0.0000 | 0.0000 | 0.0000 | - |  |
| CHU | *r* | no | no | no | no | no | no | 0.0645 | no | no | 0.1507 | no | no | no | no | no | no | no | - |  |
|  | *A* | 3.000 | 7.000 | 8.000 | 3.000 | 4.000 | 2.000 | 13.000 | 2.000 | 3.000 | 10.000 | 4.000 | 3.000 | 3.000 | 4.000 | 4.000 | 5.000 | 6.000 | 4.941 |  |
|  | *A_r_* | 3.000 | 6.666 | 7.763 | 2.625 | 3.606 | 1.998 | 12.137 | 1.863 | 2.863 | 8.547 | 3.999 | 2.994 | 2.989 | 3.998 | 3.950 | 4.440 | 5.591 | 4.649 |  |
|  | *H_o_* | 0.813 | 0.750 | 0.719 | 0.500 | 0.344 | 0.188 | 0.750 | 0.063 | 0.219 | 0.500 | 0.594 | 0.469 | 0.313 | 0.719 | 0.563 | 0.406 | 0.531 | 0.496 |  |
|  | *uH_E_* | 0.625 | 0.699 | 0.799 | 0.439 | 0.301 | 0.170 | 0.871 | 0.061 | 0.293 | 0.766 | 0.625 | 0.436 | 0.276 | 0.659 | 0.502 | 0.527 | 0.667 | 0.521 |  |
|  | *P_hw_* | 0.9936 | 0.7757 | 0.1710 | 0.8516 | 1.0000 | 1.0000 | 0.0116 | 1.0000 | 0.0078 | **0.0000** | 0.2980 | 0.7257 | 1.0000 | 0.5340 | 0.7722 | **0.0000** | 0.1172 | - |  |
|  | *P_LD_* | 0.0000 | 0.0000 | 0.0000 | 0.0000 | 0.0000 | 0.0000 | 0.0000 | 0.0000 | 0.0000 | 0.0000 | 0.0000 | 0.0000 | 0.0000 | 0.0000 | 0.0000 | 0.0000 | 0.0000 | - |  |
| GAL | *r* | no | no | no | no | no | no | no | no | no | 0.2308 | no | no | no | no | no | no | 0.1781 | - |  |
|  | *A* | 3.000 | 7.000 | 8.000 | 2.000 | 3.000 | 2.000 | 13.000 | 2.000 | 2.000 | 5.000 | 3.000 | 4.000 | 2.000 | 4.000 | 4.000 | 5.000 | 5.000 | 4.353 |  |
|  | *A_r_* | 3.000 | 7.000 | 8.000 | 2.000 | 3.000 | 2.000 | 13.000 | 2.000 | 2.000 | 5.000 | 3.000 | 4.000 | 2.000 | 4.000 | 4.000 | 5.000 | 5.000 | 4.353 |  |
|  | *H_o_* | 0.700 | 0.600 | 0.850 | 0.250 | 0.150 | 0.150 | 0.850 | 0.250 | 0.350 | 0.200 | 0.450 | 0.400 | 0.200 | 0.600 | 0.450 | 0.650 | 0.350 | 0.438 |  |
|  | *uH_E_* | 0.655 | 0.609 | 0.808 | 0.219 | 0.141 | 0.139 | 0.883 | 0.399 | 0.289 | 0.560 | 0.374 | 0.579 | 0.180 | 0.634 | 0.444 | 0.624 | 0.643 | 0.493 |  |
|  | *P_hw_* | 0.5655 | 0.5143 | 0.3457 | 1.0000 | 1.0000 | 1.0000 | 0.2916 | 0.1072 | 1.0000 | **0.0000** | 1.0000 | 0.0783 | 1.0000 | 0.2038 | 0.5834 | 0.5987 | 0.0016 | - |  |
|  | *P_LD_* | 0.0000 | 0.0000 | 0.0000 | 0.0000 | 0.0000 | 0.0000 | 0.0000 | 0.0000 | 0.0000 | 0.0000 | 0.0000 | 0.0000 | 0.0000 | 0.0000 | 0.0000 | 0.0000 | 0.0000 | - |  |
| GUR | *r* | no | no | no | no | no | no | 0.0908 | no | no | 0.1449 | no | no | no | no | no | no | no | - |  |
|  | *A* | 3.000 | 8.000 | 10.000 | 4.000 | 4.000 | 2.000 | 16.000 | 2.000 | 2.000 | 11.000 | 4.000 | 4.000 | 3.000 | 4.000 | 5.000 | 5.000 | 7.000 | 5.529 |  |
|  | *A_r_* | 3.000 | 7.490 | 8.272 | 3.095 | 3.539 | 1.997 | 12.827 | 2.000 | 2.000 | 7.973 | 3.994 | 3.673 | 2.769 | 3.834 | 4.421 | 3.946 | 5.755 | 4.740 |  |
|  | *H_o_* | 0.607 | 0.732 | 0.714 | 0.339 | 0.339 | 0.214 | 0.714 | 0.268 | 0.214 | 0.518 | 0.661 | 0.411 | 0.143 | 0.589 | 0.500 | 0.589 | 0.679 | 0.484 |  |
|  | *uH_E_* | 0.649 | 0.757 | 0.731 | 0.350 | 0.298 | 0.191 | 0.886 | 0.257 | 0.293 | 0.775 | 0.636 | 0.425 | 0.166 | 0.625 | 0.502 | 0.662 | 0.718 | 0.530 |  |
|  | *P_hw_* | 0.1945 | 0.1445 | 0.4965 | 0.4235 | 1.0000 | 1.0000 | **0.0000** | 0.7610 | 0.0560 | **0.0000** | 0.7439 | 0.6504 | 0.0573 | 0.3013 | 0.4715 | 0.0847 | 0.3852 | - |  |
|  | *P_LD_* | 0.0000 | 0.0000 | 0.0000 | 0.0000 | 0.0000 | 0.0000 | 0.0000 | 0.0000 | 0.0000 | 0.0000 | 0.0000 | 0.0000 | 0.0000 | 0.0000 | 0.0000 | 0.0000 | 0.0000 | - |  |
| BUI | *r* | no | no | no | no | no | no | no | no | no | 0.0968 | no | no | no | no | no | 0.1240 | no | - |  |
|  | *A* | 3.000 | 9.000 | 9.000 | 3.000 | 3.000 | 2.000 | 17.000 | 3.000 | 2.000 | 10.000 | 4.000 | 4.000 | 3.000 | 4.000 | 5.000 | 5.000 | 6.000 | 5.412 |  |
|  | *A_r_* | 2.999 | 8.336 | 8.201 | 2.997 | 2.741 | 1.741 | 15.292 | 2.741 | 2.000 | 8.833 | 4.000 | 3.740 | 2.936 | 3.985 | 4.726 | 4.985 | 5.599 | 5.050 |  |
|  | *H_o_* | 0.519 | 0.778 | 0.852 | 0.333 | 0.259 | 0.037 | 0.889 | 0.444 | 0.296 | 0.593 | 0.593 | 0.444 | 0.185 | 0.556 | 0.741 | 0.444 | 0.741 | 0.512 |  |
|  | *uH_E_* | 0.575 | 0.762 | 0.772 | 0.390 | 0.283 | 0.036 | 0.888 | 0.393 | 0.444 | 0.763 | 0.650 | 0.374 | 0.233 | 0.626 | 0.631 | 0.649 | 0.630 | 0.545 |  |
|  | *P_hw_* | 0.3609 | 0.4957 | 0.8948 | 0.2188 | 0.4538 | - | 0.4700 | 0.8517 | 0.0795 | 0.0055 | 0.3513 | 1.0000 | 0.0189 | 0.3414 | 0.9647 | 0.0255 | 0.9054 | - |  |
|  | *P_LD_* | 0.0000 | 0.0000 | 0.0000 | 0.0000 | 0.0000 | 0.0000 | 0.0000 | 0.0000 | 0.0000 | 0.0000 | 0.0000 | 0.0000 | 0.0000 | 0.0000 | 0.0000 | 0.0000 | 0.0000 | - |  |
| HUL | *r* | no | no | no | no | no | no | 0.0596 | no | no | 0.1049 | no | no | no | no | no | 0.2116 | no | - |  |
|  | *A* | 3.000 | 10.000 | 8.000 | 3.000 | 4.000 | 2.000 | 15.000 | 2.000 | 2.000 | 8.000 | 5.000 | 3.000 | 3.000 | 4.000 | 5.000 | 5.000 | 4.000 | 5.059 |  |
|  | *A_r_* | 3.000 | 8.357 | 7.333 | 2.943 | 3.454 | 2.000 | 13.369 | 2.000 | 1.999 | 7.275 | 4.603 | 2.943 | 2.942 | 3.979 | 4.447 | 4.936 | 3.943 | 4.678 |  |
|  | *H_o_* | 0.576 | 0.758 | 0.788 | 0.455 | 0.212 | 0.182 | 0.788 | 0.273 | 0.152 | 0.576 | 0.758 | 0.364 | 0.273 | 0.606 | 0.424 | 0.273 | 0.394 | 0.462 |  |
|  | *uH_E_* | 0.630 | 0.748 | 0.750 | 0.446 | 0.268 | 0.213 | 0.901 | 0.316 | 0.190 | 0.760 | 0.664 | 0.330 | 0.267 | 0.654 | 0.423 | 0.614 | 0.495 | 0.518 |  |
|  | *P_hw_* | 0.1948 | 0.3794 | 0.7099 | 0.5835 | 0.1120 | 0.3812 | 0.0106 | 0.3440 | 0.2979 | 0.0005 | 0.6617 | 0.7704 | 0.5508 | 0.3817 | 0.6209 | **0.0000** | 0.1313 | - |  |
|  | *P_LD_* | 0.0000 | 0.0000 | 0.0000 | 0.0000 | 0.0000 | 0.0000 | 0.0000 | 0.0000 | 0.0000 | 0.0000 | 0.0000 | 0.0000 | 0.0000 | 0.0000 | 0.0000 | 0.0000 | 0.0000 | - |  |
| FER | *r* | no | no | no | no | no | no | no | no | no | no | no | no | 0.0830 | no | no | no | no | - |  |
|  | *A* | 6.000 | 6.000 | 7.000 | 3.000 | 3.000 | 2.000 | 12.000 | 2.000 | 2.000 | 5.000 | 4.000 | 5.000 | 3.000 | 4.000 | 3.000 | 4.000 | 6.000 | 4.529 |  |
|  | *A_r_* | 5.060 | 5.377 | 6.170 | 2.212 | 2.455 | 2.000 | 10.592 | 2.000 | 2.000 | 4.606 | 4.000 | 3.667 | 2.993 | 3.979 | 2.999 | 3.212 | 5.452 | 4.046 |  |
|  | *H_o_* | 0.576 | 0.515 | 0.606 | 0.061 | 0.091 | 0.273 | 0.818 | 0.303 | 0.515 | 0.697 | 0.636 | 0.152 | 0.242 | 0.667 | 0.455 | 0.424 | 0.667 | 0.453 |  |
|  | *uH_E_* | 0.559 | 0.631 | 0.655 | 0.059 | 0.088 | 0.278 | 0.869 | 0.334 | 0.471 | 0.691 | 0.727 | 0.144 | 0.355 | 0.685 | 0.479 | 0.522 | 0.713 | 0.493 |  |
|  | *P_hw_* | 0.5415 | 0.0519 | 0.3487 | 1.0000 | 1.0000 | 0.6310 | 0.0391 | 0.4325 | 0.7964 | 0.4878 | 0.0938 | 1.0000 | 0.0146 | 0.0935 | 0.3151 | 0.1386 | 0.0060 | - |  |
|  | *P_LD_* | 0.0000 | 0.0000 | 0.0000 | 0.0000 | 0.0000 | 0.0000 | 0.0000 | 0.0000 | 0.0000 | 0.0000 | 0.0000 | 0.0000 | 0.0000 | 0.0000 | 0.0000 | 0.0000 | 0.0000 | - |  |
| Overall | PIC | 0.557 | 0.695 | 0.740 | 0.309 | 0.233 | 0.164 | 0.887 | 0.267 | 0.293 | 0.705 | 0.614 | 0.343 | 0.223 | 0.594 | 0.462 | 0.592 | 0.624 | 0.4882 |  |
|  | PID | 0.209 | 0.106 | 0.081 | 0.465 | 0.583 | 0.688 | 0.019 | 0.519 | 0.479 | 0.103 | 0.162 | 0.423 | 0.596 | 0.181 | 0.288 | 0.182 | 0.156 | 9.881×10^-12^ |  |
|  | PIsib | 0.489 | 0.414 | 0.388 | 0.693 | 0.733 | 0.832 | 0.307 | 0.725 | 0.693 | 0.405 | 0.460 | 0.669 | 0.780 | 0.466 | 0.573 | 0.468 | 0.453 | 2.977×10^-4^ |  |

Loc*,* Locations; *r*, frequency of null allele estimated using software Micro-Checker, “no” indicates no null allele detected; *A*, number of alleles; *Ar*, allele richness; *H_O_*, observed heterozygosity; *uH_E_*, unbiased expected heterozygosity; *P_HW_*, p-value for Hardy-Weinberg equilibrium test. The significance after Bonferroni correction were bolded. *P_LD_*, percentage of allele comparisons showing significant linkage disequilibrium; PIC, polymorphic information content. PID, non-exclusion probability of identity; PIsib, non-exclusion probability of sibling identity Ave., average/combined value. *****Locus in linkage disequilibrium with each other.

**Table S2**. Results of migrants’ detection analyses from GENECLASS for Swan Geese *Anser cygnoides*. Potential migrants were listed with result from first generation migrant’s detection which indicated with “a”, and results from assignment-exclusion test which indicated with “b”. Samples with consistent results were marked in italic bold. For abbreviations for the sampling locations, see Figure 1.

| Sample ID | Sampling site | Sex | GENECLASS locality of F0 detection^a^ | GENECLASS F0 migrant value [-log (L_home)]^a^ | GENECLASS F0 *P* value ^a^ | GENECLASS locality of highest probability assignment-exclusion test^b^ |
| --- | --- | --- | --- | --- | --- | --- |
| AIL7 | AIL | Male | GAL | 12.414 | 0.0056 | Unknown origin |
| UVS1 | UVS | Male | HUL | 10.528 | 0.008 | AIL/UGI/GUR/CHU/BUI/HUL |
| GUR15 | GUR | Male | CHU | 12.142 | 0.0061 | DAT/CHU |
| BEN1 | BEN | Male | CHU | 7.227 | 0 | AIL/UVS/UGI/GUR/BUS/DAT/CHU/GAL/BUI/HUL/HR/FER |
| BEN3 | BEN | Male | FER | 9.994 | 0 | AIL/UGI/DAT/BUI/FER |
| DAT9 | DAT | Male | CHU | 8.233 | 0.0023 | AIL/UGI/GUR/BEN/BUS/CHU/GAL/BUI/HUL/HR/FER |
| GAL16 | GAL | Male | HUL | 10.284 | 0 | UGI/GUR/DAT/CHU/BUI/HUL |
| BUI30 | BUI | Male | GAL | 12.864 | 0.0031 | BEN (0.05059) |
| *FER4* | FER | Male | AIL | 11.985 | 0.0043 | AIL (0.18874) |
| *FER6* | FER | Male | BEN | 12.755 | 0.0018 | BEN (0.10368) |
| AIL8 | AIL | Female | GUR | 11.541 | 0.004 | GUR/CHU/HUL |
| GUR26 | GUR | Female | CHU | 13.909 | 0.0014 | AIL (0.05633) |
| BEN2 | BEN | Female | GUR | 9.527 | 0 | AIL/GUR/BUS/CHU/GAL/BUI/HUL/FER |
| BUS8 | BUS | Female | CHU | 8.453 | 0.0017 | AIL/UGI/GUR/CHU/GAL/BUI/HUL |
| DAT5 | DAT | Female | UGI | 7.614 | 0.009 | AIL/UGI/GUR/BEN/BUS/KHT/CHU/GAL/BUI/HUL/HR/FER |
| GAL17 | GAL | Female | HUL | 12.222 | 0 | GUR/HUL |
| *HUL2* | HUL | Female | GUR | 11.773 | 0.0096 | GUR (0.11314) |
| HR1 | HR | Female | GUR | 6.352 | 0.0017 | AIL/UGI/GUR/BEN/BUS/KHT/DAT/CHU/GAL/BUI/HUL/FER |
| HR6 | HR | Female | AIL | 9.789 | 0 | AIL/GUR/BEN/KHT/CHU/GAL/BUI |
